# No Effects of Psychosocial Stress on Intertemporal Choice

**DOI:** 10.1371/journal.pone.0078597

**Published:** 2013-11-08

**Authors:** Johannes Haushofer, Sandra Cornelisse, Maayke Seinstra, Ernst Fehr, Marian Joëls, Tobias Kalenscher

**Affiliations:** 1 Department of Economics, University of Zürich, Zürich, Switzerland; 2 Department of Economics, Massachusetts Institute of Technology, Cambridge, Massachusetts, United States of America; 3 Department of Neuroscience and Pharmacology, Rudolf Magnus Institute of Neuroscience, University Medical Center Utrecht, Utrecht, The Netherlands; 4 Comparative Psychology, Institute of Experimental Psychology, Heinrich-Heine University Düsseldorf, Düsseldorf, Germany; 5 Swammerdam Institute of Life Sciences, University of Amsterdam, Amsterdam, The Netherlands; Inserm, France

## Abstract

Intertemporal choices - involving decisions which trade off instant and delayed outcomes - are often made under stress. It remains unknown, however, whether and how stress affects intertemporal choice. We subjected 142 healthy male subjects to a laboratory stress or control protocol, and asked them to make a series of intertemporal choices either directly after stress, or 20 minutes later (resulting in four experimental groups). Based on theory and evidence from behavioral economics and cellular neuroscience, we predicted a bidirectional effect of stress on intertemporal choice, with increases in impatience or present bias immediately after stress, but decreases in present bias or impatience when subjects are tested 20 minutes later. However, our results show no effects of stress on intertemporal choice at either time point, and individual differences in stress reactivity (changes in stress hormone levels over time) are not related to individual differences in intertemporal choice. Together, we did not find support for the hypothesis that psychosocial laboratory stressors affect intertemporal choice.

## Introduction

Many everyday decisions entail trading off immediate and delayed outcomes. For instance, we routinely choose between spending money today, or saving it for later consumption. In such intertemporal choices, both humans and animals tend to attach special significance to short-term rewards, a phenomenon known as “present bias” [1]–[4]. As a consequence of present bias, people frequently find it difficult to act in accordance with their own long-term interest [2], [5].

Intertemporal choices in private or professional contexts are often made under stress; managers, politicians, investment bankers, medical doctors and other professionals make vital decisions under a considerable amount of pressure. This applies, for instance, to a corporate executive who needs to trade-off the usefulness of long-term business strategies with succumbing to the pressure of reporting short-term profits, or a medical doctor who needs to decide on the spot between quick fixes relieving the symptoms of his patients, or slower but potentially more effective therapies. How stress affects intertemporal choice, though, is unknown. Here we combine theory and evidence from behavioral economics and cellular neuroscience to answer this question.

In behavioral economics, both models and evidence on intertemporal choice now distinguish between present bias on the one hand, and impatience on the other [2]. Impatience is simply the subject’s preference about consumption at different times (i.e. as soon as possible in case of high impatience) and is not irrational per se. Present bias is normatively irrational because it leads subjects to fail executing the future plans they make today and thereby reverse their preferences in favor of immediate gratification. For example, when the long term goal is to obtain a more healthy lifestyle, but you are offered a piece of your favorite pie now, you might change your preference and choose to eat the pie. However, when the pie is only available at a later point in time, you prefer your long-term goal. The application of self-control is therefore important to overcome present-bias and to stick to long term preferences, while impatient individuals consistently prefer more immediate outcomes. Here we econometrically and experimentally distinguish between these two motives, and can thus ask whether stress differentially affects present bias or impatience.

In cellular and behavioral neuroscience, it has become evident in recent years that stress affects neurobiological processes and cognitive functions in two distinct temporal domains [6]–[8]. Broadly, the picture that emerges from this research is that immediately after stress, the stress-induced changes in hormone and neurotransmitter levels facilitate short-term solutions to the stressful situation; in contrast, beginning approximately 1 h after stress onset, slower physiological changes promote restoration and future perspective after stress. More specifically, shortly after stress, corticosteroid hormones and noradrenaline synergistically promote rapid increases in neuronal activity, such as activity caused by the neurotransmitter glutamate [9], [10], the main excitatory neurotransmitter in the nervous system. Rapid effects of corticosteroids have been described for several emotion- and arousal-related brain regions such as the hippocampus and amygdala [11]. For instance, in interaction with noradrenergic activation, rapid corticosteroid actions promote amygdala-dependent processing of information [11]–[14] while higher cognitive functions mediated by e.g. the prefrontal cortex are suppressed [15]. These early physiological responses to stress likely facilitate the rapid focused attention, hypervigilance and choice of strategy required to implement the organism’s fight-or-flight response [6]; in particular, they promote habitual, reflex-like behavior at the expense of goal-directed behavior [16], which may lead to impaired behavioral control.

In contrast, the slow actions of cortisol focus on long-term restoration and future perspective after stress. Through changes in gene transcription that require 55–65 minutes to develop and last for several hours [17], stress-induced corticosteroid actions shut down the effects of noradrenaline [18]–[20] and change neuronal activity in frontal brain regions such that the stress-induced release of hormones from the pituitary is terminated [21], [22]. Behaviorally, these delayed effects of stress promote consolidation of stress-related memories for future use [6], [21], [23] and stimulate restoration of cognitive self-control [8], [24], [25]. Although these slow corticosteroid actions develop within an hour, their implications stretch well beyond this time-domain [14], [26].

Based on these previous findings, we hypothesized that this bi-directional pattern is also apparent in more complex behavioral responses carried out after stress, such as intertemporal choice. Thus, we hypothesize that intertemporal choices will be differentially affected immediately after stress versus at a later time point after stress. More specifically, we predict that immediately after stress behavioral control may be impaired and subjects would exhibit an increased propensity to choose smaller-sooner over larger-later payoffs (i.e. increased present bias), whereas we predicted the opposite result when subjects were tested at a later time point after stress.

## Materials and Methods

### Participants

142 male undergraduate students from the University of Zürich ranging in age from 19 to 29 (M = 21.97±4.23) participated in the study. We restricted our experiment to men since controlling for ovarian cycle in women is logistically difficult. Subjects were tested in two batches, spread out by a year. Because all procedures were the same between the batches and half of the subjects from each group were tested in different batches, all results were pooled. Before admission to the study, all subjects were screened in a telephone interview to exclude medication intake, somatic diseases, or any neurological or psychiatric disorders. Furthermore, psychology and economics students, self-reported heavy smokers (consumption of >5 cigarettes per day), heavy alcohol consumers (consumption of >60 g alcohol per day) and drug users were excluded. Participants were German native speakers, had not participated in a Trier Social Stress Test (TSST) before and would stay in Zürich at least for the next 12 months (for payment of their reward). The study was approved by the ethics committee of the Department of Economics at the University of Zürich and all participants provided written informed consent. Participants received a variable reimbursement for their participation, depending on the choices they made during the experiment.

### Stress Manipulation

Psychosocial stress was induced with a group version of the Trier Social Stress Test [27], [28] and involved a preparation period of 5 min, followed by a video- and audio- taped public speaking task of 12 min (a fictional job interview, see below), and a mental arithmetic task of 8 min. Both tasks were performed in front of an evaluation committee (one man and one woman both wearing white laboratory coats). We used the grouped version of the TSST (based on the procedure of Dawans et al. [28]) because it is a very efficient procedure and it elicits comparable stress responses as the original TSST [28]. Further, the grouped version of the TSST enabled the use of a control task that optimally controls for the cognitive load and circumstances of the stress task, as described below. A maximum of 4 and a minimum of 2 subjects were tested at the same time. In the job interview component of the task, participants had 3 minutes each (in the case of 4 participants) to describe why their personal qualities qualified them for a job. The committee repeatedly interrupted the presentation with questions, following a pre-prepared script. In the arithmetic task, participants were asked to count backwards in steps of 16, starting at a random 4-digit number. When a mistake was made the panel told the participant to start over. Subjects all delivered their speeches and then performed the arithmetic task. Each subject was called at least twice and in random order for every task, to induce a feeling of unpredictability. Speaking time for every participant was kept constant.

To keep the cognitive load and circumstances of the control condition as comparable as possible, only lacking the component of social evaluation, subjects in the control condition underwent the same conditions, with three important differences. First, subjects were not video- or audio- taped and there was no panel in laboratory coats; there was just a passive observer in a corner of the room. Second, the public speech was replaced by an account of what would qualify a good friend for a job. The purpose of this task was to require a similar amount of creativity and cognitive resources as the personal job interview, while not containing the same stressful element of social evaluation and having to “talk oneself up”. Finally, all subjects performed their tasks simultaneously with the other participants; this made the individual contributions unintelligible to the passive observer and the other participants, thus further reducing the social evaluative element. Total duration of the task and speaking time for each participant were matched to the parameters of the stress condition. When the speaking time for each part of the control task was finished (3 minutes for the speech and 2 minutes for the arithmetic task, in case of 4 participants), participants were asked to keep a standing position and read neutral magazines for the remaining time.

### Salivary Sampling and Biochemical Analysis

**Figure 1:**
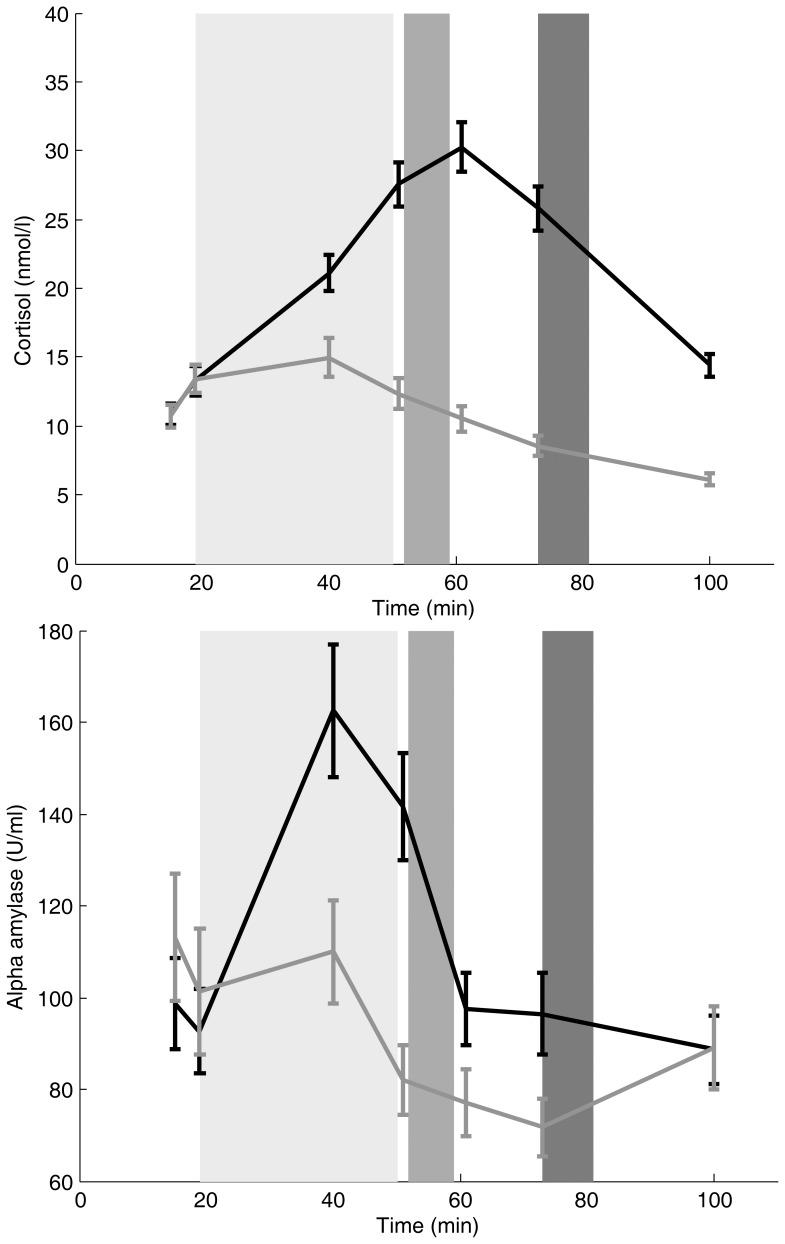
Experimental timeline and cortisol and effects of psychosocial stress on salivary cortisol (A) and alpha-amylase (B) levels. Subjects were subjected to a group-wise Trier Social Stress Test (TSST-G) or a control task, which lasted 30 minutes (light gray region). Subsequently, they performed an intertemporal choice task lasting approximately 10 min, either immediately following the TSST-G/Control task (medium gray region), or 20 min later (i.e. 55 min after onset of the stressful situation; dark grey region). The conducted ANOVAs revealed a significant Sample Period × Stress interaction for both cortisol (**A**) and alpha-amylase (**B**). *p<0.1 in post-hoc tests.

Salivary samples were obtained using Salivette sampling devices (Sarstedt, Nümbrecht, Germany) at 7 time points during the experiments (Figure 1). Salivary samples were stored at −20°C until further analysis. Free cortisol levels were measured using a commercially available immunoassay (IBL, Hamburg, Germany). Salivary alpha-amylase (sAA) levels were measured by a quantitative enzyme kinetic essay as described elsewhere [29].

### Questionnaires

Mood measurements and stress ratings were assessed shortly before and directly after the TSST-G or control task (at t = 15 and t = 50 min, see Figure 1). Subjects filled out the 10 negative affect items (rated on a 5-point scale) of the Positive and Negative Affect Scale (PANAS; [30]), resulting in a score of negative affect before and after the TSST-G or control task. At the same time points subjects rated how stressed they felt at that moment on a Visual Analogue Scale (later coded as ranging from 1 to 100). To assess impulsivity as a personality trait, subjects filled out the 30-item Barratt Impulsivity Scale (BIS; [31]) after the experimental tasks.

### Intertemporal Choice Task

Participants performed 6 blocks of an intertemporal choice task with varying delays, where decisions between a sooner smaller reward and a later larger reward were offered. In the first four blocks subjects chose between a smaller reward tomorrow, and a larger reward in a) 3 months and 1 day, b) 6 months and 1 day, c) 9 months and 1 day, and d) 12 months and 1 day. The short delay was set to “tomorrow” rather than “today” to keep transaction costs the same for sooner and later payments (see below for details on transaction costs). In the last two blocks, subjects chose between a smaller reward in 6 months and 1 day, and a larger reward in e) 9 months and 1 day, and f) 12 months and 1 day. Each block consisted of 7 binary choice trials, resulting in a total of 42 trials. The larger reward was kept constant at an amount of 40 Swiss Francs (CHF), while the sooner smaller reward started at CHF 20 and was then adjusted with a titration method according to the choices the subject made.

Titration is a standard method for identifying time preferences in the discounting literature [32]–[35]. The titration worked as follows: for each choice of the later reward, the sooner reward was increased by half the difference between it and 40 CHF; for instance, if a subject chose CHF 40 in 12 months and 1 day over CHF 20 tomorrow, the next trial would offer the subject a choice between CHF 40 in 12 months and 1 day and CHF 30 tomorrow; if the subject still chose CHF 40 in 12 months and 1 day, the next offer would be CHF 40 in 12 months and 1 day vs. CHF 35 tomorrow, and so on. For each choice of the sooner reward, the sooner reward was decreased by half of the difference between it and the previously offered soon reward. For instance, if a subject chose CHF 20 tomorrow over CHF 40 in 12 months and 1 day, the next trial would offer the subject a choice between CHF 10 tomorrow and CHF 40 in 12 months and 1 day; if the subject chose CHF 10 tomorrow, the next offer would be CHF 5 tomorrow vs. CHF 40 in 12 months and 1 day, and so on. The titration procedure lasted for 7 trials at each combination of delays; this means that each indifference point was identified to a precision of CHF 0.156 (CHF 20 *0.5^∧^7; i.e. the initial difference between CHF 20 and CHF 40/CHF 0 was halved seven times). The amount of the sooner reward at the end of this titration procedure was taken as the indifference point for the particular delay combination (i.e. the amount of the sooner smaller reward where participants switched between the smaller sooner and the later larger reward).

This procedure resulted in an individual discount function for each subject, which was used as the basis for fitting parameters of several models of intertemporal choice. In addition, we obtained a model-free measure of present bias**.** Possible serial correlation and order effects in subjects’ responses were controlled for by randomizing the order of trials across blocks (i.e. the order in which the various indifference points were determined). In addition, the side of the screen (left or right) on which the “late” and “soon” options were presented on each trial was randomized across trials.

Note that the soonest option subjects could choose in the intertemporal choice task was “tomorrow”. One may ask whether this delay can be considered small enough to be useful in identifying present bias. We chose this design for the following reasons: first, we found it difficult to include an earlier reward in the design without confounding transaction costs: the chosen option on one of the trials in the intertemporal choice task was paid out for real (i.e., participants could pick up the chosen amount on the chosen day of delivery, using a voucher valid at the University cashier’s office). If the smallest delay was “today”, choosing this option would result in lower transaction costs compared to choosing a more delayed option, because subjects are already at the University, while at any other delay than today (i.e. “tomorrow”, but also in several months) subjects may have to travel to the University specifically to pick up their payment. Therefore, in this case we would have been unable to dissociate transaction costs from present bias. Second, other forms of payment than cash vouchers proved more problematic: we judged that getting a check or cash on the day vs. receiving a check or cash in the mail later did not equate the perceived risk of the transaction; bank transfers cannot be effected on the same day and also have to be picked up at the bank before they can be consumed; Amazon vouchers cannot be turned into consumption immediately because of the delays associated with mail orders; mobile phone money transfers and pre-paid debit cards are not available in Switzerland. Thus, the “tomorrow” option seemed to us the cleanest way of eliciting time preference without risk of transaction cost confounds.

Final reimbursement consisted of a variable payment depending on participants’ choices. In particular, as was explained to the participants at the beginning of the study, one of all the choices made was randomly selected at the end of the study, and the chosen option on this trial was paid out for real (i.e., participants could pick up the chosen amount on the chosen day of delivery, using a voucher valid at the University cashier’s office).

### Procedure

Subjects were randomly assigned to one of four conditions: control-early (N = 36), control-late (N = 35), stress-early (N = 35), or stress-late (N = 36). The study was conducted between 14∶00 and 20∶00 in the (late) afternoon, when plasma cortisol levels are close to the circadian trough. Participants were instructed to refrain from smoking, eating, or drinking caffeine containing beverages at least 2h before the study, and were asked not to consume alcohol 24h before participating. An overview of the study timeline is displayed in Figure 1. Subjects were instructed not to talk to each other during the whole experiment.

Ten to fifteen minutes after subjects arrived in the laboratory, a first saliva sample was taken. Subjects were guided to a room where they received instructions and practice questions for the intertemporal choice task, to be able to administer the task directly after the stress situation without delay. Notably, subjects only received general instructions, but were not provided with any information about the actual rewards and delays during the intertemporal choice task. It is therefore unlikely that they would have decided on their choices already at this time point. When all subjects understood the task and answered the practice questions correctly, a second saliva sample was taken and a PANAS/VAS questionnaire was filled out. Next, subjects received instructions for the TSST-G or the control task, and after the 5 min preparation period participants were guided to another room, where they gave their speech. Before subjects were instructed to perform the arithmetic task, a third saliva sample was taken. Directly after the whole TSST-G or control procedure, a fourth saliva sample was taken and a further PANAS/VAS questionnaire was filled out. Next, participants were asked to sit at the chair placed behind them and, depending on the experimental condition, directly perform the intertemporal choice task on a laptop placed before them on a table (the early groups), or fill out the Barratt Impulsivity Scale and a socioeconomic questionnaire and read neutral magazines (the late groups). The total delay between the start of the TSST-G and testing of intertemporal choice in the early groups including transportation time from one room to another, biological measurements and filling out forms was 35 min. 10 min and 20 min after the end of the TSST-G or control task, the fifth and sixth saliva samples were taken, after which subjects in the late groups performed the intertemporal choice task (20 min after the early groups), and subjects in the early groups filled out the questionnaires and read magazines. After they finished, participants waited until the last saliva sample was taken 50 min after the end of the TSST-G or control task, after which they were debriefed and got their payment results (depending on the choices they made during the intertemporal decision making task), and instructions for picking up their payment.

The choice of timing of the behavioral tasks after the stress task was based on the following reasoning. The first time point was selected to target non-genomic actions of corticosteroid hormones and other rapidly acting stress hormones like noradrenaline (i.e. immediately after the TSST). At this point in time, levels of the stress hormones (including of (nor)adrenaline) are still high, so that they can evoke non-genomic actions [9], [10]; however, the time-frame is too short to allow the development of gene-mediated events. The second time point was selected such that it would just allow the development of genomic actions. Specifically, earlier findings in neurobiology show that genomic corticosteroid actions are apparent after one hour, both in the hippocampus [36] and the prefrontal cortex [22]. However, we wished to not test later than approximately one hour after stress onset in order to be as close as possible to the earlier time point, to avoid unwanted influences that cannot be controlled for, such as circadian variations in hormone level. For this reason we tested individuals between 55 and 65 minutes after onset of the TSST.

### Model Fits

For every subject and every delay level, we determined the amount at which a subject was indifferent between the sooner and the more delayed option, based on the individual indifference points (see above). This allowed us to express the subjective value of the delayed reward as a fraction of the subjective value of the immediate reward. We then plotted the relative values of the delayed rewards as a function of time. Next, for every subject, we fitted three different models to the obtained indifference points.

#### Hyperbolic discounting model

We first fitted a standard hyperbolic model [32], [34], [37]–[39] of the following shape:
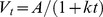
(1)where V_t_ indicates the discounted value at time t, A is the amount of reward, t the delay until reward delivery, and k is a single parameter describing the shape of the hyperbola. Because we expressed the value of the delayed rewards as a fraction of the value of the immediate reward, A = 1.

#### Exponential discounting model

Second, we fit a standard exponential discount function of the following shape:
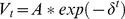
(2)where V_t_ indicates the discounted value at time t, A is the amount of reward, t is the delay until reward delivery, and δ is the parameter describing the steepness of the exponential discount function. Because we expressed the value of the delayed rewards as a fraction of the value of the immediate reward, A = 1.

#### Beta-delta quasi-hyperbolic discount model

Laibson’s beta-delta model [2] was fitted to the indifference points to obtain an estimate of the degree of impatience and present bias:
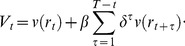
(3)where V_t_ indicates the discounted value at time t of a stream of rewards r with subjective values v as a function of time τ. This equation contains a constant, exponential discount function whose discount rate is *log (1/δ)*, thus whose steepness can be characterized by δ. The β parameter deflects the exponential discount curve and its inverse can be interpreted as the extra weight added to immediate rewards. Hence, δ can be interpreted as measuring impatience and β as measuring present bias.

The data were better described by Laibson’s quasi-hyperbolic model than the standard hyperbolic discounting model or the standard exponential model (Akaike Information Criteria, adjusting for the number of parameters: Laibson model, −11.22; hyperbolic model, −9.57; exponential model, −8.86).

#### Model-free measure of delay discounting

To obtain a model-free summary measure of discounting behavior, we computed the area under the curve (AUC) under the discount function. This was achieved by simply computing the area of each trapezoid described by adjacent pairs of indifference points and the line y = 0, and summing the areas, individually for each participant.

#### Model-free measure of present bias

Finally, to obtain a model-free measure of present bias, we proceeded as follows: present-biased subjects are those whose discounting over a given period is greater when that period is in the near future compared to when it is in the more distant future. In our design, a present-biased subject would discount more between tomorrow and 3 months and 1 day than between 3 months and 1 day and 6 months and 1 day; similarly, they would discount more between tomorrow and 6 months and 1 day than between 6 months and 1 day and 12 months and 1 day. We used this feature of our experimental design to obtain a model-free measure of present bias, without assumptions about the shape of the discount function, but computing the difference between their “tomorrow vs. 3 months and 1 days” indifference point and the “6 months and 1 day vs. 9 months and 1 day” indifference points from the intertemporal choice task; similarly, we computed the difference between their “tomorrow vs. 6 months and 1 days” indifference point and the “6 months and 1 day vs. 12 months and 1 day” indifference points. The average of these two differences is a model-free measure of present bias.

### Statistical Analysis

All measures that showed a skewed distribution with the Shapiro-Wilk test of normality (cortisol, sAA, PANAS and VAS measurements) were log-transformed and further Analyses of Variance (ANOVAs) were performed on the transformed data. Hormone measurements were analyzed using a 7 (Sample Period: t0 vs. t15 vs. t40 vs. t50 vs. t60 vs. t70 vs. t100) × 2 (Stress: TSST-G vs. Control) ×2 (Delay: Early vs. Late) General Linear Model (GLM) repeated measures ANOVA with Sample Period as a repeated measure. Subjective affect and stress measures were analyzed using a 2 (Sample Period: pre vs. post) × 2 (Stress: TSST-G vs. Control) ×2 (Delay: Early vs. Late) General Linear Model (GLM) repeated measures ANOVA with Sample Period as a repeated measure.

For our main analysis, effects of stress and timing on the indifference points of the intertemporal choice task were analyzed by a 6 (Indifference Point) × 2 (Stress: TSST-G vs. Control) × 2 (Delay: Early vs. Late) General Linear Model (GLM) repeated measures ANOVA with Indifference Points as a repeated measure. Next, effects of stress and timing were analyzed on each indifference point separately and on the model parameters with a 2 (Stress: TSST-G vs. Control) × 2 (Delay: Early vs. Late) ANOVA.

The relationship between hormone increases and stress-induced changes in discount parameters was assessed by OLS regression of model parameters on area under the curve for cortisol and sAA using heteroskedasticity-robust standard errors. Specifically, we used the following specification:
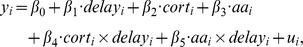
where *y_i_* is any model fit parameter from the intertemporal choice task, *cort_i_* and *aa_i_* are the area under the curve measures for cortisol and alpha-amylase, respectively, and the remaining variables are as above. This regression was run only on subjects in the stress groups.

## Results

### Stress Induction

The experimental groups did not differ on any of the measured baseline variables (cortisol, PANAS and VAS; all *p*’s >0.40). As expected, the ANOVA for cortisol showed a significant Sample Period × Stress interaction (Figure 1a, *F*_6,816_ = 68.23, *p*<0.001). Furthermore, a main effect of Sample Period (*F*_6,816_ = 68.23, *p*<0.001) and a main effect of Stress (*F*_1,136_ = 2925.19, *p*<0.001) were found. Planned simple contrasts related to baseline showed that subjects in the stress condition had increased cortisol levels from the sample taken during the TSST-G (t40) until t90 (i.e. at the end of the session; all *p*’s <0.001).

For sAA, a significant Sample Period × Stress interaction (Figure 1b, *F*_6,804_ = 18.36, *p*<0.001) and a significant main effect of Sample Period (*F*_6,804_ = 35.00, *p*<0.001) were found. Planned simple contrasts compared to baseline showed that sAA levels were increased in the stress condition from t40 (i.e. during the TSST-G) until after the TSST-G at t50 (*p*’s <0.05). Thus, the stress manipulation worked as intended, significantly raising both cortisol and alpha-amylase levels over time. Negative affect (PANAS; *F*_1,137_ = 15.60, *p*<0.001) as well as subjective stress ratings (VAS; *F*_1,137_ = 15.82, *p*<0.001) both increased in the stress group compared to the control group immediately after the stress task compared to baseline. As expected, no effects of Delay (Early vs. Late groups) were found on the stress induction measurements.

### Intertemporal Choice Performance

To assess the effect of stress on intertemporal choice, we first obtained indifference points for each of the six delay combinations used. We then fit the discounting data with three standard models, as described above: the standard hyperbolic model, the standard exponential model, and Laibson’s quasi-hyperbolic model. In the standard hyperbolic model, higher k implies greater discounting, and in the exponential model, larger rho implies greater discounting. Laibsons’ model distinguishes present bias from impatience: whereas the latter refers to the degree of discounting of future outcomes as a function of time, the former refers to extra value placed on short-term outcomes [2]. Present bias is characterized by the beta parameter, and impatience by the delta parameter. Strong present bias and strong impatience are associated with low betas and high deltas, respectively (see Materials and Methods).

The overall ANOVA on the 6 indifference points did not show a Stress × Delay interaction effect (*F*_1,138_ = 0.090, *n.s.*), nor a main effect of either Stress (*F*_1,138_ = 0.402, *n.s.*) or Delay (*F*_1,138_ = 0.011, *n.s.*) or any interaction effects with Indifference Point (all F’s <0.55, *n.s.*). ANOVAs on each indifference point individually also revealed that neither Stress, Delay nor the interaction between Stress and Delay influenced the indifference points in the intertemporal choice task (all F’s <0.71, *n.s.*) or discounting model fit parameters (all F’s <1.11, *n.s.*). Table 1 reports the descriptives and summary statistics for the indifference points and the time preference model parameters that were assessed. Thus, we found no evidence that stress affects intertemporal choice in our study. The model-free measure of discounting (i.e. the area under the curve of the discount function) was also not affected by stress; column 6 in Table 2 shows that there was neither a main effect of stress or delay, nor an interaction effect.

**Table 1:** Descriptives and summary statistics for indifference points in intertemporal choice task and discounting model fit parameters.

		Tom. vs. 3 m	Tom. vs. 6 m	Tom. vs. 9 m	Tom. vs.12 m	6 m vs.9 m	6 m vs. 12 m	Hyperbolic (k)	Exponential(delta)	Quasi-hyperbolic(delta)	Quasi-hyperbolic(beta)	Presentbias
Control, Early	mean	29.01	26.72	25.31	22.97	34.96	32.41	0.12	0.07	0.96	0.82	5.82
	S.E.	1.43	1.63	1.49	1.71	0.85	1.20	0.03	0.01	0.01	0.03	1.21
	N	36	36	36	36	36	36	36	36	36	36	36
	mean	29.22	26.65	25.86	24.46	35.26	32.57	0.14	0.08	0.96	0.83	5.98
Stress, Early	S.E.	1.42	1.76	1.91	1.95	1.10	1.56	0.05	0.02	0.01	0.03	1.35
	N	35	35	35	35	35	35	35	35	35	35	35
	mean	27.85	25.95	24.05	23.04	34.83	32.47	0.18	0.09	0.96	0.80	6.75
Control, Late	S.E.	1.62	1.94	2.10	2.19	1.12	1.19	0.04	0.02	0.01	0.04	1.16
	N	35	35	35	35	35	35	35	35	35	35	35
	mean	29.37	27.11	25.53	23.70	36.17	33.69	0.11	0.07	0.97	0.82	6.70
Stress, Late	S.E.	1.57	1.65	1.68	1.79	0.80	1.01	0.02	0.01	0.01	0.04	1.31
	N	36	36	36	36	36	36	36	36	36	36	36
Statistics Stress	F	0.328	0.096	0.315	0.316	0.715	0.301	0.429	0.526	0.106	0.287	0.002
	*p*	0.568	0.757	0.575	0.575	0.399	0.584	0.514	0.470	0.745	0.293	0.967
Statistics Delay	F	0.112	0.008	0.195	0.033	0.161	0.224	0.116	0.229	0.004	0.276	0.428
	*p*	0.739	0.927	0.660	0.857	0.689	0.636	0.734	0.633	0.951	0.600	0.514
Statistics Stress × Delay	F	0.184	0.125	0.066	0.048	0.287	0.181	1.112	0.604	0.627	0.004	0.007
	*p*	0.669	0.724	0.798	0.828	0.593	0.671	0.293	0.438	0.430	0.953	0.934

Individual rows report mean, standard errors, and number of participants for each variable, separately for each of the four experimental conditions. The F and p values of the Stress × Timing interaction effects are reported. Tom. = Tomorrow; m = months and one day.

**Table 2:** Effect of stress manipulation on time preference model parameters.

	(1) Hyperbolic (k)	(2) Exponential(delta)	(3) Quasi-hyperbolic (delta)	(4) Quasi-hyperbolic (beta)	(5) Present-bias	(6) Discount curve AUC
Stress	0.0151 (0.0587)	0.000774 (0.0209)	−0.00438 (0.0140)	0.0165 (0.0470)	0.157 (1.815)	4.666 (23.05)
Delay	0.0527 (0.0530)	0.0186 (0.0221)	−0.00687 (0.0127)	−0.0203 (0.0497)	0.930 (1.676)	−7.836 (24.57)
Stress × Delay	−0.0797 (0.0761)	−0.0230 (0.0297)	0.0149 (0.0190)	0.00411 (0.0694)	−0.210 (2.521)	10.34 (33.79)
Observations	142	142	142	142	142	142

Robust standard errors in parentheses.

We repeated the regression analysis of the discount parameters using cortisol or alpha-amylase area under the curve as independent variables using all subjects, not only those in the stress group. The results of this analysis are reported in Table 3. They reveal that subjects with higher cortisol or sAA area under the curve showed no differential discounting behavior. We did find an effect of delay- that is subjects in the delayed condition discounted more than others. This effect was attenuated in subjects with strong responses to the TSST. The results of the OLS regression analyses are summarized in tables 2–9.

**Table 3:** Effect of hormonal reactivity to stress on time preference model parameters in all subjects (stress and control group pooled).

	(1) Hyperbolic (k)	(2) Exponential (delta)	(3) Quasi-hyperbolic (delta)	(4) Quasi-hyperbolic (beta)	(5) Present-bias	(6) Discount curve AUC
Delay	0.254*** (0.006)	0.0899** (0.011)	−0.0378* (0.097)	−0.101 (0.179)	3.537 (0.240)	−61.01 (0.101)
Cortisol AUC	79.09 (0.114)	24.15 (0.159)	−4.389 (0.691)	−40.16 (0.259)	765.0 (0.540)	−8429.9 (0.615)
Delay × Cortisol AUC	−123.1** (0.026)	−42.44** (0.032)	13.10 (0.299)	74.38* (0.070)	−2052.0 (0.165)	27309.3 (0.174)
o.Delay	0 (.)	0 (.)	0 (.)	0 (.)	0 (.)	0 (.)
Alpha-Amylase AUC	3.446 (0.344)	1.257 (0.414)	−1.764 (0.159)	4.327 (0.107)	−132.8 (0.287)	−1428.1 (0.481)
Delay × Alpha-Amylase AUC	−4.597 (0.326)	−1.599 (0.422)	1.755 (0.245)	−3.761 (0.303)	78.36 (0.632)	1539.6 (0.542)
Observations	140	140	140	140	140	140

Robust standard errors in parentheses.

* *p*<0.10,

** *p*<0.05,

*** *p*<0.01.

**Table 4:** Effect of stress manipulation on indifference points in intertemporal choice task.

	(1) Tom. vs. 3 m	(2) Tom. vs. 6 m	(3) Tom. vs. 9 m	(4) Tom. vs. 12 m	(5) 6 m vs. 9 m	(6) 6 m vs. 12 m
Stress	0.217 (2.017)	−0.0758 (2.394)	0.549 (2.421)	1.491 (2.588)	0.302 (1.394)	0.154 (1.968)
Delay	−1.153 (2.157)	−0.777 (2.532)	−1.259 (2.576)	0.0714 (2.773)	−0.131 (1.404)	0.0600 (1.695)
Stress × Delay	1.297 (3.024)	1.234 (3.495)	0.927 (3.618)	−0.833 (3.832)	1.044 (1.957)	1.067 (2.512)
Observations	142	142	142	142	142	142

Robust standard errors in parentheses.

**Table 5:** Effect of hormonal reactivity to stress on time preference model parameters in the Stress groups.

	(1) Hyperbolic (k)	(2) Exponential (delta)	(3) Quasi-hyperbolic (delta)	(4) Quasi-hyperbolic (beta)	(5) Present-bias	(6) Discount curve AUC
Delay	0.291* (0.059)	0.106** (0.049)	−0.0440 (0.258)	−0.169 (0.208)	3.654 (0.510)	−97.20* (0.097)
Cortisol AUC	103.3 (0.225)	28.34 (0.315)	−6.394 (0.741)	−35.22 (0.513)	−665.8 (0.699)	−8161.5 (0.757)
Delay × Cortisol AUC	−147.2* (0.094)	−51.56* (0.083)	12.54 (0.535)	117.2* (0.052)	−1123.4 (0.574)	46016.2 (0.116)
o.Delay	0 (.)	0 (.)	0 (.)	0 (.)	0 (.)	0 (.)
Alpha-Amylase AUC	3.943 (0.422)	1.346 (0.479)	−2.474 (0.191)	6.493** (0.047)	−196.1 (0.166)	−1822.8 (0.481)
Delay × Alpha-Amylase AUC	−3.612 (0.527)	−1.136 (0.635)	2.424 (0.270)	−7.524 (0.122)	−7.752 (0.970)	715.5 (0.834)
Observations	70	70	70	70	70	70

Each column represents an OLS regression; the dependent variable is the column title, and the independent variables are shown as rows. They include i. an indicator variable for the delay condition (“Delay”), ii. Cortisol area under the curve for each individual subject in the Stress groups (minutes × nmol/L, divided by 1,000,000 for ease of readability), and iii. Alpha-amylase area under the curve for each individual subject in the Stress groups. The cells show the OLS regression coefficients, betas, which are to be interpreted such that a one-unit change in the independent variable is associated with a beta change in the dependent variable. P-values based on heteroskedasticity-robust standard errors are shown in parentheses. Asterisks denote statistical significance of the individual coefficients: *p<0.10,

** *p*<0.05.

**Table 6:** Effect of stress manipulation on indifference points in intertemporal choice task for patient subjects only.

	(1) Tom. vs. 3 m	(2) Tom. vs. 6 m	(3) Tom. vs. 9 m	(4) Tom. vs. 12 m	(5) 6 m vs. 9 m	(6) 6 m vs. 12 m
Stress	−0.596 (1.819)	−0.152 (1.525)	3.184** (1.227)	1.656 (1.590)	0.378 (0.907)	0.809 (0.808)
Delay	−0.448 (1.922)	0.0822 (1.579)	2.429* (1.417)	2.038 (1.936)	1.290* (0.698)	0.245 (0.780)
Stress × Delay	2.419 (2.365)	0.812 (2.111)	−3.019 (1.880)	−2.941 (2.522)	−1.854 (1.128)	−1.755 (1.223)
Observations	71	71	71	71	71	71

Robust standard errors in parentheses.

* *p*<0.10,

** *p*<0.05,

*** *p*<0.01.

**Table 7:** Effect of stress manipulation on indifference points in intertemporal choice task for impatient subjects only.

	(1) Tom. vs. 3 m	(2) Tom. vs. 6 m	(3) Tom. vs. 9 m	(4) Tom. vs. 12 m	(5) 6 m vs. 9 m	(6) 6 m vs. 12 m
Stress	0.104 (2.551)	−1.238 (2.811)	−3.271 (2.859)	−0.0474 (2.948)	−0.257 (2.277)	−1.293 (3.092)
Delay	−2.874 (2.489)	−2.930 (2.941)	−6.194** (2.881)	−3.375 (2.657)	−2.114 (2.087)	−0.889 (2.427)
Stress × Delay	1.492 (3.730)	3.392 (4.017)	6.644* (3.818)	3.252 (4.016)	4.680 (3.159)	4.961 (3.894)
Observations	71	71	71	71	71	71

Robust standard errors in parentheses.

* *p*<0.10,

** *p*<0.05.

**Table 8:** Effect of stress manipulation on patient responding in intertemporal choice task.

	(1) Tom. vs. 3 m	(2) Tom. vs. 6 m	(3) Tom. vs. 9 m	(4) Tom. vs. 12 m	(5) 6 m vs. 9 m	(6) 6 m vs. 12 m
Stress	−0.0321 (0.0402)	−0.00703 (0.0503)	0.0431 (0.0504)	0.0336 (0.0489)	0.0118 (0.0565)	0.0142 (0.0580)
Delay	−0.0158 (0.0407)	−0.0111 (0.0474)	0.0757 (0.0498)	0.0785 (0.0541)	−0.00454 (0.0546)	−0.0185 (0.0573)
Stress × Delay	0.0399 (0.0600)	0.0340 (0.0698)	−0.0434 (0.0724)	−0.112 (0.0749)	0.0126 (0.0777)	0.00828 (0.0799)
Observations	142	142	142	142	142	142

Robust standard errors in parentheses.

**Table 9:** Effect of stress on inconsistent responses in time preference task.

	Inconsistent
Stress	0.0764 (0.183)
Delay	0.0407 (0.421)
Stress × Delay	−0.0962 (0.198)
Observations	142

Robust standard errors in parentheses.

One possible explanation for the lack of an effect of stress on discounting observed here is that subjects’ responses in the discounting task may have been at floor or ceiling. To address this concern, first note from the minimum and maximum discount parameters reported in Table 10 that subjects in fact exhibited a broad range of discount behavior, making this alternative account prima facie unlikely. To address the concern more explicitly, we repeated our main analysis separately for patient vs. impatient subjects. Specifically, we performed a median split on the mean of the six indifference points for each subjects, and then regressed the indifference points of each of these groups individually on the stress and delay indicator variables. The results are reported in Tables 6 and 7. They are not qualitatively or quantitatively different from our main findings (i.e. neither patient nor impatient participants exhibited an effect of stress on discounting).

**Table 10:** Summary statistics for indifference points in intertemporal choice task and discounting model fit parameters.

		Tom. vs. 3 m	Tom. vs. 6 m	Tom. vs. 9 m	Tom. vs. 12 m	6 m vs. 9 m	6 m vs. 12 m	Hyperbolic (k)	Exponential(delta)	Quasi-hyperbolic (delta)	Quasi-hyperbolic (beta)	Presentbias	Discountcurve AUC
Control, Early	Mean	29.01	26.72	25.31	22.97	34.96	32.41	0.12	0.07	0.96	0.82	5.82	335.12
	S.E.	1.43	1.63	1.49	1.71	0.85	1.20	0.03	0.01	0.01	0.03	1.21	15.15
	Min	9.84	7.66	4.84	2.34	19.84	19.84	0	0	0.87	0.34	−9.38	115.31
	Max	40	40	38.59	38.91	40	40	0.91	0.37	1	1	27.66	468.98
	N	36	36	36	36	36	36	36	36	36	36	36	36
Stress, Early	Mean	29.22	26.65	25.86	24.46	35.26	32.57	0.14	0.08	0.96	0.83	5.98	339.79
	S.E.	1.42	1.76	1.91	1.95	1.10	1.56	0.05	0.02	0.01	0.03	1.35	17.38
	Min	9.84	0.16	0.78	1.09	13.91	4.84	0	0	0.61	0.26	−11.72	71.72
	Max	40	40	40	39.53	40	40	1.65	0.49	1	1	24.53	479.3
	N	35	35	35	35	35	35	35	35	35	35	35	35
Control, Late	Mean	27.85	25.95	24.05	23.04	34.83	32.47	0.18	0.09	0.96	0.80	6.75	327.29
	S.E.	1.62	1.94	2.10	2.19	1.12	1.19	0.04	0.02	0.01	0.04	1.16	19.35
	Min	7.66	0.16	0.16	0.16	10.16	19.53	0	0	0.72	0.24	−0.16	133.13
	Max	40	40	40	40	40	40	0.84	0.34	1	1	29.38	480
	N	35	35	35	35	35	35	35	35	35	35	35	35
Stress, Late	Mean	29.37	27.11	25.53	23.70	36.17	33.69	0.11	0.07	0.97	0.82	6.70	342.3
	S.E.	1.57	1.65	1.68	1.79	0.80	1.01	0.02	0.01	0.01	0.04	1.31	15.36
	Min	9.22	8.59	4.84	0.16	21.41	14.84	0	0	0.87	0.27	−6.88	146.72
	Max	40	40	39.53	39.22	40	40	0.54	0.28	1	1	25.16	475.08
	N	36	36	36	36	36	36	36	36	36	36	36	36

Individual rows report mean, standard errors, minimum and maximum values, and number of participants for each variable, separately for each of the four experimental conditions.

Null-results are difficult to interpret since the lack of significance can be due to a lack of effect, or due to an insufficiently small sample size and thus insufficient power. To this end, we conducted a power analysis using G-Power [40]. The power analysis revealed that a group size of 142 subjects should have been sufficient to detect even small effects sizes of 0.2 standard deviations with a power of 0.8. Thus, although our results provide no ultimate evidence for the absence of effect, it is unlikely that the lack of significance is due to insufficient statistical power.

We next asked whether individual differences in stress responsivity might obscure any effects of our stress manipulation on discounting behavior. To answer this question, we regressed the model parameters on individual cortisol and alpha-amylase stress reactivity, for the stress group only, with an interaction term for delay (see Table 5). We found a weakly significant positive relationship between stress-induced alpha-amylase increases and beta. Thus, in strong alpha-amylase stress responders, stress may decrease present bias somewhat; conversely, people with blunted stress responses would exhibit stress-induced increases in present bias. Overall, however, we observed no significant effects of stress and timing of stress on intertemporal choice.

To further investigate the possibility that stress may have affected the path individual participants took to reach the indifference point, we conducted an additional analysis in which we did not use the indifference points, but the proportion of patient responses of each participant as the outcome variable, taking into account all responses of each participant from the first choice situation until an indifference point was reached. The results are reported in Table 8. Again none of the coefficients was significant, suggesting that when the proportion of patient responses is used as the outcome variable instead of the indifference points, the results do not change. A possible confound to our results is that subjects’ responses to the time preference questions may be inconsistent, and that this inconsistency may be affected by stress. This is an important concern, since it has been shown that, for example, the effect of working memory load on discounting can be explained by increased randomness in responses under higher working memory load, rather than truly different time preferences [41], [42]. In principle, this mechanism could underlie our results as well, in that it might obscure any effects of stress on intertemporal choice.

To assess whether our subjects showed inconsistent responses in the time preference task, and whether these responses were affected by stress, we analyzed whether our subjects showed non-monotonic discount functions, - that is whether any indifference point identified by our titration algorithm at a particular delay was *lower* than any indifference point at a later delay (e.g., we identified indifference points as inconsistent if a subject was indifferent between 20 CHF in one day and 40 CHF in 6 months, and 30 CHF in one day and 40 CHF in 9 months). Note that our experimental design did not make it possible for subjects to give inconsistent responses when identifying any *particular* indifference point, since the indifference points were approximated by a titration algorithm. Thus, any given indifference point, by the nature of the algorithm, was obtained through choices that were “consistent” by definition. However, mistakes that subject made during the titration would manifest themselves in inconsistencies between indifference points; this is what we analyzed in the following.

Overall, 20.77% of indifference points showed evidence of inconsistency. However, the proportion of inconsistent responses for a particular subject was not dependent on whether this subject was in the stress or control, or early or late conditions. In an OLS regression with the percentage of inconsistent indifference points as the dependent variable, and stress and delay, and their interaction, as the independent variables, no coefficient was significant (Stress: beta = 0.0764, p = 0.183; Delay: beta = 0.0407, p = 0.421; Stress × Delay: beta = −0.0962, p = 0.198). Thus, stress does not appear to have affected response consistency in the intertemporal choice task.

Note that we used consistency here to refer to noise in the respondent’s choices, rather than to preference reversals or hyperbolic discounting. We deem a set of indifference points inconsistent if at least one indifference point is higher than another indifference point from the same set that corresponds to a shorter time horizon. For instance, a set of indifference points would be considered inconsistent if CHF 40 were discounted to CHF 30 over 3 months, but to CHF 35 over 6 months. Importantly, because the slope of such a discount function is positive over some interval, this sense of inconsistency is distinct from hyperbolic discounting, which implies a negative slope over the entire support of the discount function. This sense of inconsistency is therefore not captured by models of hyperbolic discounting or time inconsistency, such as Laibson’s beta-delta model [2] or Takahashi’s q-exponential discounting [43].

As an alternative measure of choice consistency, it might be tempting to turn to the rate of convergence of the titration procedure. However, this rate of convergence cannot be distinguished from preference, and therefore cannot serve as a measure of choice consistency: if a participant has a defined indifference point for a given pair of delays, then there is only one path through the titration procedure that leads to that indifference point. The rate at which the procedure’s current best estimate of the indifference point changes is therefore determined by the distance of the participant’s true indifference point from the starting point of the titration procedure. As an example, suppose a subject is presented with a choice of CHF 20 tomorrow vs. CHF 40 in 3 months at the beginning of the titration procedure; assume further that their true indifference point for this pair of delays is CHF 30. The 6 trials of the procedure allow the participant to approximate their true indifference point up to +/−15 cents. If the participant does this, the procedure will converge at an average rate of CHF 10/6 = CHF 1.67 per trial. In contrast, if their true indifference point were CHF 38, the procedure would converge at a rate of CHF 3 per trial. Thus, the rate of convergence is a function of the distance of the indifference point from the starting point of the titration procedure, and can therefore not be used as an estimate of choice consistency. Put differently, the titration procedure takes each decision by the participant at face value and ignores the fact that some of these decisions may be affected by noise. This will lead to noisily estimated indifference points; however, as shown in Table 9, we can estimate the degree of noise in the indifference points, and find that it is not affected by experimental condition.

## Discussion

We here investigated the effect of stress on intertemporal choice. We had hypothesized, based on theory and evidence from behavioral economics and cellular neuroscience, that immediately after stress subjects would exhibit an increased propensity to choose smaller-sooner over larger-later payoffs, whereas we predicted the opposite result when subjects were tested 20 minutes after the end of the stress test. Recent evidence from cellular and behavioral neuroscience suggests that shortly after stress, individuals turn to simple behavioral strategies. For instance, humans exposed to a psychosocial stressor use a simpler (striatal) stimulus-response rather than a more complex spatial learning strategy [44]. Individuals also shift from goal-directed to habitual control in instrumental behavior shortly after stress [16]. Underlying biological mechanisms of these rapid stress effects are thought to involve both catecholamines and corticosteroid hormones [7], [8], [16], [44], [45], the latter probably accomplishing non-genomic actions [9]. Conversely, recent evidence suggests that the later effects of stress may serve the function of normalizing the stress response and preparing the organism for the future [8], [24], [25]; consistent with this view is the finding that slow genomic corticosteroid effects improve spatial memory formation in mice [23] and e.g. contextualization of emotional information and working memory in humans [25], [46].

However, our results show no evidence for an effect of stress on intertemporal choice at either time point, suggesting that intertemporal choice may in general not be affected by psychosocial laboratory-induced stress. We also found no evidence that stress-induced changes in hormone levels (cortisol and alpha-amylase) correlate with individual differences in intertemporal choice, suggesting that these stress responses may not substantially contribute to the observed intertemporal choice behavior.

Our findings contrast sharply with a growing body of recent evidence suggesting that stress may affect decision-making. Keinan [47] found that subjects were impaired in a verbal analogy task when they were threatened with uncontrollable compared to controllable electric shocks. Gray [48] found that subjects made suboptimal decisions in a temporally extended choice task when the task was presented in a negative emotional compared to a neutral context. Van den Bos et al. [49] and Preston et al. [50] reported that performance on the Iowa Gambling Task was impaired under stress, particularly in men. Finally, Porcelli & Delgado [51] induced stress using the cold-pressor task [52], in which subjects immerse their hand in ice-cold water, and found that this stress induction increased the reflection effect in risky decision-making: stressed subjects showed stronger risk aversion in the domain of gains, and stronger risk seeking in the loss domain. Although timing was not specifically addressed in any of these studies, the design was such that rapid rather than delayed effects of stress were targeted. Our study sought to extend these previous findings on risky choice into the intertemporal domain. However, we did not find any effects of stress on intertemporal choice, independent of the timing of choice relative to stress onset. This finding does not support the view that stress strongly affects all domains of decision-making; instead it suggests that some aspects of decision-making may be susceptible to the effects of stress, while others may not.

An alternative explanation for the found null-results might be an interaction between an increased preference for more immediate rewards and a stress induced reduction in reward responsiveness [53]–[55]. A general decrease in hedonic or motivational value of rewards could manifest in increased choice for delayed reinforcers, provided that temporal discounting is not constant and reward to utility mapping is not linear, but follows a concave utility function [56], [57]. A decrease in reward responsiveness might therefore have counteracted increased present bias directly after stress induction. Speculatively, a similar interaction occurring in the late condition, leading to zero net effects, possibly indicates that a longer timeframe is necessary to dissociate early versus late effects of stress on inter-temporal decision making.

Of course, when understanding how stress affects decision making it is important to keep in mind that there are individual differences in reactivity to social stress. For example, social stress can either lead to “challenging states”, when individuals believe they have the personal resources to cope, or “threat states”, when situational demands are perceived to outweigh resources [58]. These individual differences in responses to stress might explain part of the somewhat inconsistent data on stress effects on decision making. One recent study [59] investigated the role of anticipatory stress on delay discounting, and found that trait perceived stress and the orientation of the stress task (present-oriented vs. future-oriented speech) interact with stress to affect delay discounting. These factors were not investigated in the current study and may partly explain the lack of effects.

A further possibility as to how our null finding may be explained rests in the particular paradigms used here, in particular the use of the TSST to induce psychosocial stress, and the titration procedure to obtain discount parameters. For instance, it is conceivable that the stress condition of the TSST, in which participants find themselves under close scrutiny by a panel of intimidating judges, leads participants to behave in a “more desirable” fashion; in the context of a discounting task, this might lead to more patient responding than would otherwise result from being stressed. Similarly, the abstract nature of our time discounting task may not be adequate for picking up an effect of stress on impulsive responding, which may be better captured with a task that allows for “hot” responses. Future work might therefore attempt to use stress induction methods which do not put participants under social pressure, such as the Cold Pressor task; and discounting paradigms which better capture impulsive responding than the present titration task.

Furthermore, in the current study the titration procedure of the intertemporal choice task might have triggered response strategies and limited the assessment of effects of stress on choice consistency. In addition, the different delays of reward choices were limited, with the soonest reward delay being tomorrow rather than today. Having the soonest reward tomorrow instead of today could in particular have weakened the effect of stress on the measure of present bias. Further studies with random choices, as well as immediately available reward options, are necessary to shed light on these issues.

It remains possible that particular aspects of our experimental design were the source of the lack of an effect of stress on choices. The studies discussed before tested both men and women, and two of them [49], [50] found clear gender differences. In our study only male subjects were tested, which limits our conclusion that psychosocial stress does not affect intertemporal choice only to male subjects. Regarding the timing of the stressor, it should be mentioned that the late group was tested on the intertemporal choice task at a time point where presumable genomic cortisol effects are just starting to develop. Based on animal studies, these genomic effects start to develop 55–65 minutes after the onset of stress [17], [18], which is the exact time interval after which the intertemporal choice task is given. Since it is not known if genomic effects need a comparable time to develop in humans, and cortisol might take longer to reach elevated levels after stress, we cannot exclude that genomic effects might not have fully developed when testing the late group. Moreover, cortisol levels were still elevated in the late group during the intertemporal choice task, so non-genomic actions might also have played a role here, therefore precluding a clear distinction between genomic and non-genomic actions of cortisol in the “late” group. Future studies may need to explore different time scales, varying the delay between stress onset and the task, as well as reward delays within the intertemporal choice task, to fully understand the complexity of the effects of stress and stress hormones on intertemporal choice.

In conclusion, while previous studies have shown that decision-making is strongly susceptible to environmental and somatic factors, such as individuals’ responses to stress and variations in hormonal balance, the present study suggests instead that some aspects of decision-making may be more stable than suggested by this literature.
